# Correlation between the AKI classification and outcome

**DOI:** 10.1186/cc7123

**Published:** 2008-11-20

**Authors:** Marlies Ostermann, Rene Chang, The Riyadh ICU Program Users Group

**Affiliations:** 1 Departments of Critical Care and Nephrology, Guy's & St Thomas' Foundation Hospital, Westminster Bridge Road, London SE1 7EH, UK; 2 Department of Nephrology & Transplantation, St George's University Hospital, Blackshaw Road, London SW17 0QT, UK

## Abstract

**Introduction:**

The Acute Kidney Injury Network proposed a new classification for acute kidney injury (AKI) distinguishing between three stages. We applied the criteria to a large intensive care unit (ICU) population and evaluated the impact of AKI in the context of other risk factors.

**Methods:**

Using the Riyadh Intensive Care Program database, we applied the AKI classification to 22,303 adult patients admitted to 22 ICUs in the UK and Germany between 1989 and 1999, who stayed in the ICU for 24 hours or longer and did not have end-stage dialysis dependent renal failure.

**Results:**

Of the patients, 7898 (35.4%) fulfilled the criteria for AKI (19.1% had AKI I 3.8% had AKI II and 12.5% had AKI III). Mortality in the ICU was 10.7% in patients with no AKI, 20.1% in AKI I, 25.9% in AKI II and 49.6% in AKI III. Multivariate analysis confirmed that AKI III, but not AKI I and AKI II, were independently associated with ICU mortality (odds ratio (OR) = 2.27). Other independent risk factors for ICU mortality were age (OR = 1.03), sequential organ failure assessment (SOFA) score on admission to the ICU (OR = 1.11), pre-existing end-stage chronic health (OR = 1.65), emergency surgery (OR = 2.33), mechanical ventilation (OR = 2.83), maximum number of failed organ systems (OR = 2.80) and non-surgical admission (OR = 3.57). Cardiac surgery, AKI I and renal replacement therapy were associated with a reduced risk of dying in the ICU. AKI II was not an independent risk factor for ICU mortality. Without renal replacement therapy as a criterion, 21% of patients classified as AKI III would have been classified as AKI II or AKI I. Renal replacement therapy as a criterion for AKI III may inadvertently diminish the predictive power of the classification.

**Conclusions:**

The proposed AKI classification correlated with ICU outcome but only AKI III was an independent risk factor for ICU mortality. The use of renal replacement therapy as a criterion for AKI III may have a confounding effect on the predictive power of the classification system as a whole.

## Introduction

There is increasing agreement that universal criteria for acute kidney injury (AKI) are needed to facilitate research and progress in the field of acute renal failure [1-3]. In 2002, the Acute Dialysis Quality Initiative (ADQI) workgroup convened an international interdisciplinary group that ultimately proposed the RIFLE classification for AKI, which distinguished between risk, injury, failure, loss and end-stage kidney disease [2]. These criteria have now been applied to more than 70,000 patients with varying acute problems and chronic comorbidities. All studies showed an increase in mortality with worsening RIFLE class. In a systematic review of 13 studies, Ricci and colleagues concluded that there was a clear correlation between the RIFLE classification and outcome [4]. The pooled estimate of relative risk (RR) for death increased from risk (RR = 2.4) to injury (RR = 4.15) to failure (RR = 6.37) compared with non-AKI.

In 2004, the ADQI group and representatives from three nephrology societies established the Acute Kidney Injury Network (AKIN) [3]. Its intentions are to facilitate international, interdisciplinary and intersocietal collaborations and to ensure progress in the field of AKI, including the development of uniform standards for the definition and classification of AKI. As part of this process, the RIFLE nomenclature and classification was modified to a staging/classification system differentiating between AKI stage I, II and III. In addition, a 48-hour time window for the diagnosis of AKI was introduced to ensure that the process was acute (Table 1).

**Table 1:** Definition and classification/staging system for acute kidney injury (AKI)*.

**AKI stage**	**Creatinine criteria**	**Urine output criteria**
**AKI stage I**	Increase of serum creatinine by ≥ 0.3 mg/dl (≥ 26.4 μmol/L) or increase to ≥ 150% – 200% from baseline	Urine output < 0.5 ml/kg/hour for > 6 hours

**AKI stage II**	Increase of serum creatinine to > 200% – 300% from baseline	Urine output < 0.5 ml/kg/hour for > 12 hours

**AKI stage III**	Increase of serum creatinine to > 300% from baseline or serum creatinine ≥ 4.0 mg/dl (≥ 354 μmol/L) after a rise of at least 44 μmol/L or treatment with renal replacement therapy	Urine output < 0.3 ml/kg/hour for > 24 hours or anuria for 12 hours

Diagnostic criteria for AKI includes an abrupt (within 48 hours) reduction in kidney function defined as an absolute increase in serum creatinine of either 0.3 mg/dl or more (≥ 26.4 μmol/L) or a percentage increase of 50% or more (1.5 fold from baseline) or a reduction in urine output. *according to Mehta and colleagues [3]

Using the AKI classification, Barrantes and colleagues found that 31.5% of 496 patients in a medical intensive care unit (ICU) met the criteria for AKI. Hospital mortality was significantly higher in patients with AKI than in those without (45.8% versus 25.7%) [5]. Bagshaw and colleagues applied the AKI criteria to 120,123 critically ill patients during the first 24 hours after admission to the ICU and compared them with AKI as defined by the RIFLE classification. They concluded that the AKI criteria did not improve the sensitivity and predictive ability of classifying AKI in the first 24 hours in the ICU [6].

The aim of our study was to apply the AKI criteria to a large ICU population during the entire stay in the ICU and to evaluate the impact of AKI in the context of other risk factors.

## Materials and methods

### Study population

We retrospectively analysed The Riyadh Intensive Care Program database which contains demographic and daily physiological data of 41,972 adult patients admitted to 19 ICUs in the UK and three ICUs in Germany between June 1989 and October 1999. The AKI classification is based on changes in serum creatinine within a 48 hour period, treatment with renal replacement therapy (RRT) or reduction in urine output. Our database does not include any creatinine values pre-admission to the ICU, so we decided to include only patients who had at least two creatinine results taken on different days while in the ICU, that is, we only included patients who stayed in the ICU for 24 hours or more or had treatment with RRT on the first day in the ICU. In addition, we excluded 797 patients with pre-existing dialysis dependent end-stage renal failure (ESRF) and three patients with incomplete data. The remaining 22,303 patients were included in the study.

### Data analysis

The AKI criteria were applied to 22,303 patients. Due to a lack of data on 6- or 12-hourly urine volumes, we only used the creatinine criteria to determine the AKI categories. The creatinine criteria describe changes in renal function without specifying the direction of change. As pointed out by experts in the field (personal communication), these creatinine changes could describe two scenarios: AKI based on a progressive rise in creatinine or AKI based on a high creatinine that subsequently falls to baseline. In order to identify patients with AKI based on rising, as well as falling, creatinine values and to incorporate the 48-hour time window for the diagnosis of AKI, we constructed a computer program that compared each day's creatinine value with subsequent values in the following two days until death or discharge from ICU (i.e. day 1 creatinine would be compared with days 2 and 3, then day 2 value compared with days 3 and 4, etc). [see Additional data files 1 and 2]. Depending on the change, patients were classified as having no AKI, AKI I, AKI II or AKI III. The most severe degree of AKI was recorded as the final AKI stage.

Acute severity was measured using the Acute Physiology and Chronic Health Evaluation (APACHE) II and Sequential Organ Failure Assessment (SOFA) scoring system [7]. Organ system failures were assessed according to the method proposed by Knaus and colleagues [8], supplemented by a definition for gastrointestinal failure (failure to tolerate enteral nutrition) [9]. The highest number of failed organs (excluding AKI) on any day during the stay in the ICU was recorded as 'maximum number of associated organ failure'. The local ethics committee was not involved and the need for informed consent was waived because the study required neither an intervention nor breach of privacy or anonymity.

### Statistical analysis

The statistical package SPSS (Version 14.0, Woking, UK) was used for all statistical analyses. Continuous variables were expressed as mean ± standard deviations (SD) and 95% confidence intervals (CI) or median and range. Student's t-tests, chi-square tests, Fisher's exact tests and Mann-Whitney tests were employed in univariate analyses to evaluate statistical significance (p < 0.05). Multivariate logistic regression analysis was conducted to identify independent predictors of all-cause ICU mortality and to obtain odds ratios (ORs). Variables that were found to be significant risk factors in univariate analyses (p < 0.05) were entered simultaneously in the multivariable model (enter method). These variables included 10 categorical variables (gender, presence of pre-existing end-stage chronic illness, mechanical ventilation, AKI categories, RRT, emergency surgery, elective surgery, non-surgical admission, haemoglobin (Hb) <9 g/dl on admission to the ICU and admission after cardiac surgery) and three numerical variables (age, SOFA score and maximum number of associated organ failure). ORs were estimated from the b coefficients obtained, with respective 95% CIs. Calibration of the model was assessed using the Hosmer-Lemeshow goodness-of-fit test. Discrimination capability was evaluated by determination of the area under the receiver operating characteristics (ROC) curve.

## Results

### Incidence of AKI and outcome

Among all 22,303 patients, 7898 (35.4%) fulfilled the criteria for AKI (Table 2). Of those, 19.1% had AKI I, 3.8% fulfilled criteria for AKI II and 12.5% had AKI III. According to the AKI classification, 14,405 patients (65.6%) had no evidence of AKI. In general, AKI III occurred later after admission to the ICU compared with AKI I. On the day when the criteria for maximum AKI stage were fulfilled, 24% of patients with AKI III had failure of three or more other organs compared with 6.4% of patients with AKI II and 3.4% of patients with maximum AKI I. Similarly, 79.9% of patients with AKI III were ventilated on the day when they fulfilled the criteria for AKI III compared with 67.4% of patients with AKI II and 69.4% of patients with maximum AKI I.

**Table 2:** Characteristics and outcome depending on degree of renal function.

	**No AKI** **n = 14,405** **(65.6%)**	**AKI 1** **n = 4259** **(19.1%)**	**AKI II** **n = 857** **(3.8%)**	**AKI III** **n = 2782** **(12.5%)**	**AKI** **(I + II + III)** **n = 7898** **(35.4%)**	**p** **AKI** **vs no AKI**	**p** **AKI III** **vs** **AKI I**
**Age**							
Mean (SD)	60.5 (16.56)	62.1 (15.93)	60.4 (16.55)	61.1 (15.66)	61.5 (15.91)	< 0.0001	0.089
Range	16 to 99	16 to 95	16 to 92	16 to 90	16 to 95		
**APACHE II score at admission to ICU**							
Median	13	16	17	21	18	< 0.0001	< 0.0001
Range	0 to 49	0 to 44	3 to 39	1 to 52	0 to 52		
**SOFA score on admission to ICU**							
Median	5	7	7	9	7	< 0.0001	< 0.0001
Range	0 to 21	0 to 18	2 to 19	0 to 22	0 to 22		
Number of associated failed organs at admission to ICU*							
Median	0	1	1	1	1	< 0.0001	< 0.0001
Range	0 to 6	0 to 5	0 to 5	0 to 6	0 to 6		
Maximum number of associated failed organ systems*							
Median	1	1	2	2	2	< 0.0001	< 0.0001
Range	0 to 6	0 to 5	0 to 6	0 to 6	0 to 6		
**Mechanical ventilation**							
Number of patients	8379 (58.2%)	3441 (80.8%)	696 (81.2%)	2416 (86.8%)	6553 (82.97%)	< 0.0001	< 0.0001
**Outcome**							
ICU mortality	1548 (10.7%)	856 (20.1%)	222 (25.9%)	1379 (49.6%)	2457 (31.1%)	< 0.0001	< 0.0001
Hospital mortality	2438 (16.9%)	1272 (29.9%)	307 (35.8%)	1610 (57.9%)	3189 (40.4%)	< 0.0001	< 0.0001
**Length of stay in ICU (days): ICU survivors**							
Median	2	6	7	9	7	< 0.0001	< 0.0001
Range	(2 to 55)	(3 to 109)	(3 to 112)	(1 to 270)	(1 to 270)		
**Length of stay in ICU (days): ICU non-survivors**							
Median	2	7	7	8	7	< 0.0001	< 0.0001
Range	(2 to 64)	(3 to 73)	(3 to 54)	(1 to 110)	(1 to 110)		
**Timing of AKI in ICU**							
First 48 hours	-	3529 (82.9%)	558 (65.1%)	2075 (74.6%)	6162 (78.0%)		
Day 3 to 5	-	424 (9.96%)	120 (14.0%)	332 (11.9%)	876 (11.1%)		
Day 6 to 9	-	193 (4.5%)	68 (7.9%)	207 (7.4%)	468 (5.9%)		
Day 10 – 14	-	73 (1.7%)	49 (5.7%)	99 (3.6%)	221 (2.8%)		
Day 15 – 19	-	20 (0.5%)	20 (2.3%)	31 (1.1%)	71 (0.9%)		
> 19^th ^day	-	20 (0.5%)	42 (4.9%)	38 (1.4%)	100 (1.3%)		

* excluding acute kidney injury

AKI = acute kidney injury; APACHE = Acute Physiology and Chronic Health Evaluation; ICU = intensive care unit; SD = standard deviation; SOFA score = Sequential Organ Failure Assessment score.

**Figure 1:**
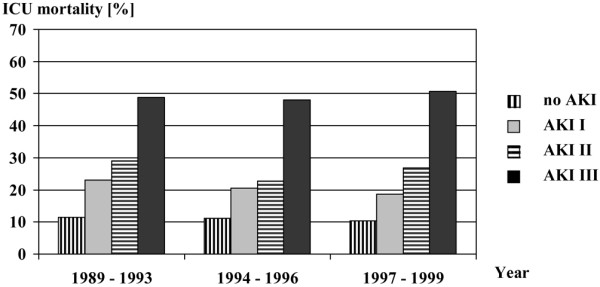
**Outcome of AKI over time**. AKI = acute kidney injury; ICU = intensive care unit.

Any degree of AKI was associated with a significantly increased all-cause ICU and hospital mortality compared with not having AKI (Table 2). Without controlling for any other risk factors, the OR for death in the ICU was 2.59 for patients with AKI I, 3.24 for patients with AKI II and 9.38 for patients with AKI III compared with not having AKI (p < 0.0001). There was no significant change in outcome in the different AKI stages over time (Figure 1). The AKI classification correlated with length of ICU admission: median length of stay was shortest among patients without AKI and rose with increasing severity of AKI (Table 2).

### Impact of confounding factors

**Figure 2:**
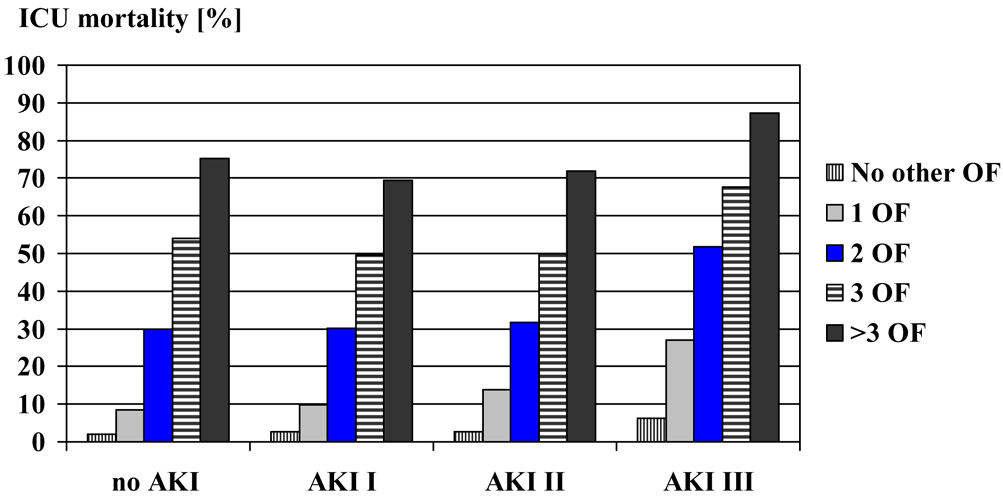
**Impact of associated maximum organ failure**. AKI = acute kidney injury; ICU = intensive care unit; OF = number of maximum associated failed organ systems.

Patients with AKI had a higher APACHE II score, SOFA score and more failed organ systems on admission to the ICU when compared with patients without AKI (Table 2). In all AKI categories, ICU mortality rose with increasing number of other failed organ systems (Figure 2). In patients with the same maximum number of failed organs, outcome was worst in patients with AKI III. There was no significant difference between AKI I and AKI II patients with the same number of associated failed organ systems. The proportion of patients who needed mechanical ventilation during their stay in the ICU increased from 58.2% among patients without AKI to 80.8% in patients with AKI I and 81.2% in patients with AKI II to 86.8% among patients with AKI III (Table 2).

Among the group of patients with AKI III, 1847 patients (64.3%) received RRT (Tables 3 and 4) of whom 1728 patients (93.6%) were treated with continuous arterio-venous haemofiltration or continuous veno-venous haemo(dia)filtration alone or in combination with intermittent haemodialysis or peritoneal dialysis. The remaining patients had intermittent haemodialysis alone or in combination with peritoneal dialysis. In a univariate analysis, both, ICU and hospital outcome were worse in patients treated with RRT compared with patients with AKI III who did not have RRT (ICU mortality 54.1% versus 40.6%; hospital mortality 61.6% versus 50.5%) (Table 3). However, patients treated with RRT were generally sicker, as evidenced by a higher APACHE II score and SOFA score on admission to ICU, more associated organ failure and a higher proportion on mechanical ventilation.

**Table 3:** Differences between AKI III on RRT and AKI III without RRT.

**Factor**	**AKI III** **on RRT** **(n = 1847)**	**AKI III** **without RRT** **(n = 935)**	**p**
**Male gender**	1242 (67.2%)	660 (70.6%)	0.08
**Age**			
Mean (SD)	60.09 (15.68)	63.14 (15.42)	0.28
**APACHE II on admission to ICU**			
Median (range)	22 (1 to 52)	19 (2 to 46)	< 0.0001
Mean (SD)	22.92 (7.80)	19.84 (6.63)	
**SOFA score on admission to ICU**			
Median (range)	10 (0 to 22)	7 (0 to 17)	0.0063
Mean (SD)	10.23 (3.18)	7.61 (2.96)	
Organ failure on admission to ICU*			
Median (range)	2 (0 to 6)	1 (0 to 4)	< 0.0001
Mean (SD)	1.72 (1.20)	1.11 (0.95)	
Maximum organ failure during ICU*			
Median (range)	2 (0 to 6)	2 (0 to 6)	< 0.0001
Mean (SD)	2.27 (1.17)	1.67 (0.97)	
**Mechanical ventilation**	1687 (91.3%)	729 (77.97%)	< 0.0001
**Pre-existing chronic illness**	469 (25.4%)	238 (25.5%)	0.97
**Haemoglobin ≤ 9 g/dl on admission to the ICU**	496 (26.9%)	193 (20.6%)	0.0004
**Cardiac surgery**	236 (12.8%)	95 (10.2%)	0.051
**Mortality**			
ICU mortality	999 (54.1%)	380 (40.6%)	< 0.0001
Hospital mortality	1138 (61.6%)	472 (50.5%)	< 0.0001

* excluding acute kidney injury

AKI = acute kidney injury; APACHE = Acute Physiology and Chronic Health Evaluation; ICU = intensive care unit; RRT = renal replacement therapy; SD = standard deviation; SOFA score = Sequential Organ Failure Assessment score.

**Table 4:** Characteristics of ICU survivors and non-survivors (univariate analysis).

**Characteristics**	**ICU** **Survivors** (n = 18,298)		**ICU** **Non-survivors** (n = 4005)		**Odds ratio** **(95% CI)**	**p**
			
	**n**	**%**	**N**	**%**		
Male gender	11,265	61.6	2441	60.9	0.97 (0.91 to 1.05)	0.48
Mean age in years (95% CI)	60.3 (60.1 to 60.6)		63.5 (63.0 to 63.95)			< 0.0001
Median APACHE II score at admission to ICU (range)	13 (0 to 49)		21 (0 to 52)			< 0.0001
Median SOFA score on admission to ICU (range)	5 (0 to 20)		8 (0 to 22)			< 0.0001
**Degree of maximum renal dysfunction**						
No AKI	12,857	70.3	1,548	38.7	0.55 (0.53 to 0.57)	< 0.0001
AKI I	3403	18.6	856	21.4	1.15 (1.08 to 1.23)	< 0.0001
AKI II	635	3.5	222	5.5	1.60 (1.38 to 1.85)	< 0.0001
AKI III	1403	7.7	1379	34.4	4.49 (4.20 to 4.80)	< 0.0001
**Type of admission**						
Non-surgical	9236	50.5	2873	71.7	2.49 (2.31 to 2.68)	< 0.0001
Elective surgery	6328	34.6	477	11.9	0.26 (0.23 to 0.28)	< 0.0001
Emergency surgery	2734	14.9	655	16.4	1.11 (1.01 to 1.22)	0.026
**Source of admission**						
Operating room	8561	46.8	1072	26.8		
Emergency room	3413	18.7	750	18.7		
Ward (including HDU)	3845	21.0	1574	39.3		
Hospital transfers	1894	10.4	531	13.3		
Recovery room	543	3.0	62	1.5		
Other	42	0.2	16	0.4		
**Chronic end-stage diseases**						
Present	3225	17.6	1,148	28.7	1.88 (1.74 to 2.03)	< 0.0001
**Haemoglobin on admission to ICU**						
Haemoglobin ≥ 9 g/dl	15,846	86.6	3238	80.8		
Haemoglobin < 9 g/dl	2452	13.4	767	19.2	1.53 (1.40 to 1.67)	< 0.0001
Cardiac surgery *						
Admission post cardiac surgery	2507	13.7	244	6.1	0.41 (0.36 to 0.47)	< 0.0001
**Mechanical ventilation**						
Ventilated	11,274	61.6	3658	91.3	6.57 (5.86 to 7.36)	< 0.0001
**Renal replacement therapy**						
RRT for AKI	848	4.6	999	24.9	5.38 (4.94 to 5.86)	< 0.0001
**Number of failed organs on day of admission to ICU**						
0 failed organ	9210	50.3	704	17.6	0.21 (0.19 to 0.23)	< 0.0001
1 failed organ	6561	35.9	1455	36.3	1.02 (0.95 to 1.10)	0.58
2 failed organs	2068	11.3	1164	29.1	3.22 (2.96 to 3.49)	< 0.0001
≥ 3 failed organs	459	2.5	682	17.0	7.98 (7.05 to 9.03)	< 0.0001
**Maximum number of associated organ failures during entire ICU stay (including AKI)**						
0 failed organ	5653	30.9	96	2.4	0.05 (0.04 to 0.07)	< 0.0001
1 failed organ	6110	33.4	459	11.5	0.26 (0.23 to 0.29)	< 0.0001
2 failed organs	4053	22.1	993	24.8	1.16 (1.07 to 1.26)	0.0003
3 failed organs	1933	10.6	1350	33.7	4.31 (3.97 to 4.67)	< 0.0001
> 3 failed organs	549	3.0	1107	27.6	12.35 (11.07 to 13.78)	< 0.0001

* coronary artery bypass surgery and/or valve surgery.

AKI = acute kidney injury; APACHE = Acute Physiology and Chronic Health Evaluation; CI = confidence interval; HDU = high dependency unit; ICU = intensive care unit; RRT = renal replacement therapy; SOFA score = Sequential Organ Failure Assessment score.

### Multivariate analysis

In a multivariate analysis, non-surgical admission, mechanical ventilation, maximum number of associated organ failure, admission after emergency surgery and AKI III were the strongest independent risk factors for ICU mortality, followed by pre-existing end-stage chronic illness, SOFA score on admission to ICU and age (Table 5). In contrast, male gender, AKI II and Hb < 9 g/dL on admission to ICU were not independently associated with ICU mortality. Admission after cardiac surgery, AKI I and RRT were associated with a reduced risk of mortality in the ICU. The area under the ROC curve was 0.88 (Hosmer-Lemeshow chi-square = 106.478; eight degrees of freedom, p < 0.0001).

**Table 5:** Multivariate logistic regression analysis: risk factors for ICU mortality.

**Variables**	**B**	**p**	**OR**	**95% CI**
Admission post cardiac surgery	-0.567	0.000	0.567	0.47 to 0.69
RRT for AKI	-0.202	0.039	0.817	0.674 to 0.99
Age	0.024	0.000	1.025	1.02 to 1.03
SOFA score on admission to ICU	0.104	0.000	1.109	1.09 to 1.13
Pre-existing chronic diseases	0.499	0.000	1.647	1.49 to 1.82
Renal function				
No AKI				
AKI I	- 0.024	0.000	0.82	0.73 to 0.91
AKI II	0.051	0.59	1.05	0.87 to 1.27
AKI III	0.820	0.000	2.27	1.92 to 2.69
Admission after emergency surgery	0.846	0.000	2.329	1.997 to 2.72
Maximum number of failed organ systems in ICU	1.028	0.000	2.795	2.66 to 2.94
Ventilation	1.040	0.000	2.828	2.48 to 3.23
Non-surgical admission	1.273	0.000	3.572	3.12 to 4.09
Constant	-5.589	0.000	0.004	

AKI = acute kidney injury; CI = confidence interval; ICU = intensive care unit; OR = odds ratio; RRT = renal replacement therapy; SOFA score = Sequential Organ Failure Assessment score.

### Additional factors

#### Timing and progression of AKI

There was a significantly higher ICU mortality in patients in whom the diagnosis of AKI was based on a rising creatinine level compared with AKI patients with an initial raised creatinine level that later decreased (Table 6). Similarly, outcome was worse in AKI III patients who gradually progressed from AKI I or AKI II to AKI III compared with patients with AKI III without prior decline.

**Table 6:** Progression of AKI/

**AKI progression**	**Number of patients**	**ICU mortality**	**p**
**AKI I **(n = 4259)			
with rising creatinine values	1995 (46.8%)	522 (26.2%)	< 0.0001
with falling creatinine values	2264 (53.2%)	334 (14.8%)	
**AKI II **(n = 857)			
with rising creatinine values	439 (51.2%)	157 (35.8%)	< 0.0001
with falling creatinine values	418 (48.8%)	65 (15.6%)	
**AKI III **(n = 2782)			
with rising creatinine values	639 (23%)	318 (49.8%)	< 0.0001
with falling creatinine values	296 (10.6%)	62 (20.9%)	
AKI III based on RRT criteria	1847 (66.4%)	999 (54.1%)	
AKI I/AKI II progressing to AKI III	1664 (59.8%)	796 (47.8%)	p = 0.029
AKI III without prior AKI I/AKI II	1118 (40.2%)	583 (46.2%)	

AKI = acute kidney injury; ICU = intensive care unit; RRT = renal replacement therapy.

#### Impact of RRT as a criterion for AKI III

The definition of AKI III includes 'treatment with RRT', which is a subjective criterion. We examined how many patients with AKI III would have been categorised differently if RRT had not been used as a criterion. Of a total of 2782 AKI III patients, 1847 had RRT of whom only 573 patients fulfilled the creatinine criteria for AKI III. Of the remaining group, 691 patients were oliguric with a urine output < 400 ml/day and would have (probably) fulfilled the urine criteria for AKI III. The remaining 583 patients had RRT without a 300% change in creatinine or a creatinine ≥ 354 μmol/L (as per AKI III criteria) or without being oliguric. Their ICU mortality was 43.4%. If RRT had not been included as a criterion for AKI III, these patients would have been classified as having AKI II, AKI I or even 'no AKI' which would have changed the overall mortality in these groups.

## Discussion

To date, all proposed classifications for AKI (RIFLE criteria [2] and differentiation between acute renal injury, acute renal failure and acute renal failure syndrome [10]) have demonstrated that the risk of death is higher in patients with a worse degree of AKI, independent of how AKI is defined [4,11]. Our data show that the same applied to the AKI classification: ICU mortality was higher in patients with different degrees of AKI compared with patients without AKI. However, in contrast to the RIFLE classification, only AKI III was independently associated with ICU mortality.

The validity of any classification system for AKI depends on whether it clearly differentiates between normal renal function and AKI, as well as between different grades of severity. The exact cut-off criteria need to be objective and prognostic but should also be easy to ascertain. The RIFLE classification meets some of these standards but not all [12,13]. The latest AKI staging system represents an improvement: it has a lower cut-off for AKI (i.e. rise in serum creatinine by ≥ 0.3 mg/dl or 26.4 μmol/L) based on data by Chertow and colleagues [14] and no longer uses 'estimated glomerular filtration rate' as a criterion but only serum creatinine and urine output. Other important changes from the RIFLE classification are the inclusion of a 48-hour window for the diagnosis of AKI and the decision to classify patients on RRT automatically as having AKI III.

Despite its correlation with outcome, this new classification is still not perfect. Firstly, the criteria for AKI III are a mixture of creatinine values, urine results and a therapeutic intervention (i.e. RRT). Due to the lack of universal guidelines for RRT (when to start, which type of RRT to use in what dose and when to stop), RRT is a completely subjective criterion, dependent on individual decision making. In our study, 31.6% of all patients on RRT had a rise in creatinine of less than 300% and a urine output of more than 400 ml/24 hours when RRT was started. These patients were only classified as having AKI III because of RRT. The clinical but subjective decision for or against RRT can automatically change the AKI category. Although patients with AKI III on RRT had a worse outcome than AKI III patients not treated with RRT (Table 3), in a multivariate analysis, RRT was independently associated with a reduced risk of mortality in the ICU. Without clear consensus on the practice of RRT, these limitations of the proposed AKI classification are difficult to overcome unless a marker of renal function can be identified that is not affected by any therapeutic interventions.

A time frame is clearly important for the diagnosis of AKI. The AKI network chose a 48-hour window which will ensure that identified cases are definitely 'acute'. However, a narrow window like this may miss patients with progressive renal dysfunction in whom the creatinine level rises steadily but never by more than 0.3 mg/dl (26.4 μmol/L) or 150% in 48 hours and never beyond 354 μmol/L, the criteria for AKI III. In our analysis, 2014 patients classified as having no AKI, had serum creatinine levels of more than 140 mol/L, 316 patients even had serum creatinine values of more than 270 μmol/L. Although it is possible that they may have had a degree of pre-existing chronic kidney disease, it is also possible that they had AKI without the necessary changes in serum creatinine within the required time period. A one-week time frame was previously suggested by the ADQI group in the original RIFLE criteria. Using the AKI classification with a seven-day instead of a 48-hour time frame, we found a higher incidence of AKI (39.5% instead of 34.4%; AKI I 19.3%, AKI II 6.7% and AKI III 13.5%). ICU mortality would have altered only slightly (AKI I 21.0%, AKI II 24.9%, AKI III 49.0%).

Our results confirm that AKI in ICU patients is not only a very common problem (34.4%) but also correlates with significant mortality. AKI III was associated with almost the same risk of death in the ICU as having emergency surgery. Although loss of renal function results in metabolic and physiological derangements and is often associated with other chronic and acute comorbidities, our analysis shows that the worse prognosis of AKI III was independent of other organ failures or being ventilated. Clermont and colleagues also previously suggested that the increased mortality of patients with AKI was not simply due to loss of renal function *per se *and that other, so far unknown, factors were responsible [15]. They compared 254 ICU patients with AKI as defined by graded changes in serum creatinine with 57 ICU patients with dialysis-dependent ESRF. They showed that patients treated with either continuous venovenous haemodialysis or intermittent haemodialysis for AKI had an ICU mortality rate four-times higher than ESRF patients. At first glance, our data would confirm this: ICU mortality among patients with AKI III treated with RRT was significantly higher than that of the excluded 797 patients with ESRF (53.8% versus 20.8%; p < 0.0001). However, patients with AKI III were generally sicker as evidenced by more associated organ failure and a higher proportion of patients on mechanical ventilation (91.3% versus 60.9%), which makes it difficult to compare these two groups.

The reason why only AKI III, and not AKI I or AKI II, were independent risk factors for death in the ICU is not clear. A possible explanation may be related to the use of RRT as a criterion: 583 patients received RRT but did not fulfil the creatinine or urine creatinine level for AKI III. Without using RRT as a criterion, these patients would have been classified as having AKI I or II, which would have altered the outcome in these groups. Further work will be necessary to explain this observation. Hopefully, with ongoing research and work by the AKI network we may be able to gain more insight into the complexities and confounding factors of AKI.

To the best of our knowledge, this is the largest study on the epidemiology of AKI during the entire ICU stay as defined by the newly proposed AKI classification. It is important to consider its strengths and limitations. The retrospective nature is a weakness, especially because we did not have any pre-ICU admission data, including previous creatinine results. However, it would take a long time to validate any new classification system in such a large patient group (> 20,000 patients in > 20 ICUs) prospectively. Secondly, it is also possible that we may have underestimated the exact incidence of AKI by excluding patients who stayed in the ICU for less than 24 hours. In addition, we only used creatinine levels obtained during the stay in the ICU and may have missed patients with serum creatinine values higher than previous baseline levels but no subsequent change in renal function while in the ICU. Finally, due to the lack of 6- and 12-hour urine results in our database we may have also missed patients who would have fulfilled the AKI criteria on urine results only. Therefore, the true incidence of AKI in the ICU may have been higher than 34.4%. It is also worth acknowledging that our conclusions are based on data from a 10-year period before 2000. Although we didn't find any significant changes in outcome between 1989 and 1999, it is possible that they have occurred since.

A major strength of this work is its size and comprehensive analysis. The results are based on a large cohort of more than 20,000 patients who were heterogeneous in terms of baseline demographics, acute medical problems and comorbidities, which makes them representative of a wide ICU population. Our conclusions are strengthened by the fact that we controlled for several important risk factors as part of the validation process.

## Conclusion

The proposed classification for AKI correlated with ICU and hospital outcome but only AKI III was an independent risk factor for ICU mortality. The introduction of a 48-hour time frame for the diagnosis of AKI may miss patients with a slow but progressive decline in renal function. RRT as a criterion for AKI III is not objective and may have had a confounding effect on the predictive power of the classification system as a whole.

## Key messages

• There is a correlation between the newly proposed classification for AKI and outcome in the ICU but only AKI III was an independent risk factor for ICU mortality.

• In a multivariate analysis, non-surgical admission, mechanical ventilation, maximum number of associated organ failure, admission after emergency surgery and AKI III were the strongest independent risk factors for ICU mortality.

• The introduction of a 48-hour window for the diagnosis of AKI may miss patients with a slow but progressive acute decline in renal function.

• RRT as a criterion for AKI III may have a confounding effect on the predictive power of the AKI classification as a whole.

## Abbreviations

ADQI: Acute Dialysis Quality Initiative; AKI: acute kidney injury; AKIN: Acute Kidney Injury Network; APACHE: Acute Physiology and Chronic Health Evaluation; CI: confidence interval; ESRF: end stage dialysis dependent renal failure; ICU: intensive care unit; OR: odds ratio; ROC: receiver operating characteristics; RIFLE: Risk, Injury, Failure, Loss, End stage classification for acute kidney injury; RR: risk ratio; RRT: renal replacement therapy; SD: standard deviation; SOFA: sequential organ failure assessment.

## Competing interests

The authors declare that they have no competing interests.

## Authors' contributions

RC is in charge of the Riyadh Intensive Care Program database. Both authors extracted the data from the database and performed the analyses. MO wrote the draft and RC provided critiques. Both authors approved the final manuscript.

## Supplementary Material

Additional file 1 a word file containing a description of the computer algorithm used to identify patients with AKI. Click here for file

Additional file 2 a word file containing two tables that illustrate in detail how the computer algorithm works. Click here for file
