# Supplementary material for: Correlation between the AKI classification and outcome
Source: Crit Care. 2008 Nov 20;12(6):R144. doi: 10.1186/cc7123 (PMC2646305; doi:10.1186/cc7123)
Supplement: Additional file 1 — a word file containing a description of the computer algorithm used to identify patients with AKI. [file cc7123-S1.doc]

**Computer algorithm to identify patients with Acute Kidney Injury**

The definitions of the criteria to classify patients as having Acute Kidney Injury (AKI) Stage I, II or III require for the serum creatinine value of a particular day in the Intensive Care Unit (ICU) to be compared with those over the next 48 hours, i.e. the next 2 consecutive values. (Please refer to Appendix Table 1 for further explanation and illustration).

The computer algorithm has to be able to do this for patients with any number of days in the ICU. (For example, patient 1 [Appendix Table 1, Rows 1 to 15; Appendix Table 2 Rows 1 to 7] has 7 days’ stay and patient 2 [Appendix Table 1, Row 16] has only 1 day stay and patient 3 [Appendix Table 1, Rows 17 to 19] has 3 days’ stay). For patient 1 who was in ICU for 7 days, the algorithm has to be able to compare the day 5 serum creatinine value (90.91) against those of days 6 and 7 [Appendix Table 1, Rows 13 to 15]. Having reached the end, it has to be able to scan the next patient and start the process again.

The algorithm to scan through the daily serum creatinine concentrations of ICU patients in order to classify them into the various stages of Acute Kidney Injury would include the following steps:

1. All patients admitted with APACHE II diagnosis of end stage chronic renal failure are excluded.
2. For each remaining patient, the number of days in ICU is calculated.
3. A set of arrays is declared to hold the daily values of key information. The number of elements for each array variable is the number of records (days in the ICU). In the case of Patient 1 the number of elements are 7.
4. The array elements are populated with the daily values of serum creatinine and other key information, such as organ system failures (not shown) for the patient for the particular admission episode. The corresponding serum creatinine values (AValue) for each ICU day for patients 1 and 2 are shown in Appendix Table 2 and the values of column AValue for Rows 1, 2, 3, 6, 9, 12, 15 and Rows 20, 21, 22 and 25.
5. The serum creatinine and other allied data from the first ICU day are used to populate a set of Reference variables defined with the pre-fix “current variable”. The value of the relevant serum creatinine is held in the variable Cvalue and the corresponding ICU day in the variable Cday. When Cday is equal to Aday, the values of CValue and AValue are the same. (See columns 5,6,7 and 8 of Row 1)
6. A set of counters are initialised such that the variable ADay is reset after the passage of two days. The day in ICU is used to identify which of the array elements will be used in the comparisons. Thus, AValue[3] holds the serum creatinine for day 3, if the ICU Day is 3, the serum creatinine for the day 3 is 154 μmol/L,([Row 3], Columns 4,6 and 8) and so on.
7. The counters have been set to change such that if the day of the Reference record (Cday) is 1, the values of the serum creatinine to be compared will be held in the array elements for the next 48 hours, which would be days 1, 2 and 3. The relevant array elements will be held in Avalue[1], Avalue[2] and Avalue[3].
8. Cvalue (for day 1) would be the same value as Avalue[1], It would be compared against Avalue[2] and Avalue[3] (Rows 1 to 3 of Appendix Table 1).
9. If Avalue[2] or Avalue[3] exceed Cvalue, it is a case of rising serum creatinine values (Row 3, Column 10). Conversely, if Cvalue exceeds Avalue[x], it is a case of falling creatinine values (Row 8, Column 9).
10. After every 48 hours, the counters are reset. For the second cycle, Cvalue takes on the value of Avalue[2] (Row 4, Columns 5 and 7). The new Cvalue (Avalue[2]) is compared against Avalue[3],and Avalue[4] (Rows 5 and 6). The same is done for subsequent cycles after every 24 hours.
11. If the change in serum creatinine meets the criteria for AKI Stage I, II or III, the patient is classified as having the corresponding AKI stage (shaded rows). The higher of the two serum creatinine and all associated variables such as the status of the various organ systems are stored. (In Row 3, the serum creatinine stored would be 154 μmol/L.) The date of AKI will be the date associated with the value of the higher creatinine.
12. After classifying all patients within the database into AKI Stages I - III based on changes in the serum creatinine concentration, the entire table is scanned once again. This time, if a patient received renal replacement therapy whilst in ICU, the previous AKI stage would be over written to Stage III.
13. When the last comparison has been done for a particular episode, the algorithm proceeds to examine the records of the next patient of the series. (Appendix Table 1, Rows 16, 17, and 20)
14. For the purpose of this paper, all patients with ICU stay of only 1 day or with pre existing dialysis dependent renal failure were excluded. This manoeuvre reduced the number of patients from 41,972 to 22,303.
