# Supplementary material for: Correlation between the AKI classification and outcome
Source: Crit Care. 2008 Nov 20;12(6):R144. doi: 10.1186/cc7123 (PMC2646305; doi:10.1186/cc7123)
Supplement: Additional file 2 — a word file containing two tables that illustrate in detail how the computer algorithm works. [file cc7123-S2.doc]

**Appendix Table 1 Illustration of computer algorithm to identify patients with acute kidney injury**

| **Row** | **Patient** | **AKI Stage** | **ICU day** | **Cday** | **Aday** | **CValue** | **AValue** | **CV-AV** | **CV/AV** | **Type** |
| --- | --- | --- | --- | --- | --- | --- | --- | --- | --- | --- |
| 1 | 1 | 0 | 1 | 1 | 1 | 100 μmol/L | 100 μmol/L | 0 | 1 |  |
| 2 | 1 | 0 | 2 | 1 | 2 | 100 μmol/L | 100 μmol/L | 0 | 1 |  |
| 3 | 1 | 1 | 3 | 1 | 3 | 100 μmol/L | 154 μmol/L | -54.55 | 0.6 | As |
| 4 | 1 | 0 | 2 | 2 | 2 | 100 μmol/L | 100 μmol/L | 0.00 | 1.00 |  |
| 5 | 1 | 1 | 3 | 2 | 3 | 100 μmol/L | 154 μmol/L | -54.55 | 0.6 | As |
| 6 | 1 | 0 | 4 | 2 | 4 | 100 μmol/L | 90.91 μmol/L | 9.09 | 1.1 |  |
| 7 | 1 | 0 | 3 | 3 | 3 | 154.55 μmol/L | 154.55 μmol/L | 0.00 | 1 |  |
| 8 | 1 | 1 | 4 | 3 | 4 | 154.55 μmol/L | 90.91 μmol/L | 63.64 | 1.7 | Des |
| 9 | 1 | 1 | 5 | 3 | 5 | 154.55 μmol/L | 90.91 μmol/L | 63.64 | 1.7 | Des |
| 10 | 1 | 0 | 4 | 4 | 4 | 90.91 μmol/L | 90.91 μmol/L | 0 | 1 |  |
| 11 | 1 | 0 | 5 | 4 | 5 | 90.91 μmol/L | 90.91 μmol/L | 0 | 1 |  |
| 12 | 1 | 0 | 6 | 4 | 6 | 90.91 μmol/L | 90.91 μmol/L | 0 | 1 |  |
| 13 | 1 | 0 | 5 | 5 | 5 | 90.91 μmol/L | 90.91 μmol/L | 0 | 1 |  |
| 14 | 1 | 0 | 6 | 5 | 6 | 90.91 μmol/L | 90.91 μmol/L | 0 | 1 |  |
| 15 | 1 | 0 | 7 | 5 | 7 | 90.91 μmol/L | 90.91 μmol/L | 0 | 1 |  |
| 16 | 2 | 0 | 1 | 1 | 1 | 90.91 μmol/L | 90.91 μmol/L | 0 | 1 |  |
| 17 | 3 | 0 | 1 | 1 | 1 | 90.91 μmol/L | 90.91 μmol/L | 0 | 1 |  |
| 18 | 3 | 0 | 2 | 1 | 2 | 90.91 μmol/L | 90.91 μmol/L | 0 | 1 |  |
| 19 | 3 | 1 | 3 | 1 | 3 | 90.91 μmol/L | 154.55 μmol/L | -63.64 | 0.6 | Asc |
| 20 | 4 | 0 | 1 | 1 | 1 | 100 μmol/L | 100 μmol/L | 0 | 1 |  |
| 21 | 4 | 0 | 2 | 1 | 2 | 100 μmol/L | 118.18 μmol/L | -18.18 | 0.8 |  |
| 22 | 4 | 0 | 3 | 1 | 3 | 100 μmol/L | 109.09 μmol/L | -9.09 | 0.9 |  |
| 23 | 4 | 0 | 2 | 2 | 2 | 118.18 μmol/L | 118.18 μmol/L | 0 | 1 |  |
| 24 | 4 | 0 | 3 | 2 | 3 | 118.18 μmol/L | 109.09 μmol/L | 9.09 | 1.1 |  |
| 25 | 4 | 1 | 4 | 2 | 4 | 118.18 μmol/L | 90.91 μmol/L | 27.27 | 1.3 | Des |

Abbreviations: AKI = acute kidney injury; ICU = intensive care unit; Aday = assessment day; AV = AValue = creatinine value on assessment day; Cday = corresponding reference day; CV = CValue = corresponding reference value (with which the creatinine of a particular day will be compared); As = AKI with ascending creatinine values; Des = AKI with descending creatinine values

**Appendix Table 2 Serum creatinine values for 2 patients with acute kidney injury**

| **Pat** | **ICU Day (Aday)** | **Creatinine (AValue)** |
| --- | --- | --- |
| 1 | 1 | 100 μmol/L |
| 1 | 2 | 100 μmol/L |
| 1 | 3 | 154 μmol/L |
| 1 | 4 | 90.91 μmol/L |
| 1 | 5 | 90.91 μmol/L |
| 1 | 6 | 90.91 μmol/L |
| 1 | 7 | 90.91 μmol/L |
| 4 | 1 | 100 μmol/L |
| 4 | 2 | 118.18 μmol/L |
| 4 | 3 | 109.09 μmol/L |
| 4 | 4 | 90.91 μmol/L |

Abbreviations: Pat = patient; ICU = intensive care unit; Aday = assessment day; AValue = creatinine value on assessment day
